# lncRNA Mirt1: A Critical Regulatory Factor in Chronic Intermittent Hypoxia Exaggerated Post-MI Cardiac Remodeling

**DOI:** 10.3389/fgene.2022.818823

**Published:** 2022-02-09

**Authors:** Xinxia Wang, Zexuan Li, Yunhui Du, Yuanyuan Xing, Yingying Guo, Yushi Zhang, Ruifeng Guo, Wei Gong, Shaoping Nie, Xiao Wang

**Affiliations:** ^1^ Department of Respiratory and Critical Care Medicine, Beijing Anzhen Hospital, Capital Medical University, Beijing, China; ^2^ Center for Coronary Artery Disease, Department of Cardiology, Beijing Anzhen Hospital, Capital Medical University, Beijing, China; ^3^ Beijing Institute of Heart, Lung and Blood Vessel Diseases, Beijing Anzhen Hospital, Capital Medical University, Beijing, China; ^4^ Comprehensive Ultrasound Department, Beijing Anzhen Hospital, Capital Medical University, Beijing, China

**Keywords:** chronic intermittent hypoxia, lncRNA, myocardial infarction, competing endogenous RNA, RNA-sequencing

## Abstract

Chronic intermittent hypoxia (CIH) is the main feature of obstructive sleep apnea (OSA) and is known to exaggerate cardiac remodeling after myocardial infarction (MI). However, the specific contribution of CIH to overall OSA-induced pathological complications and the transcriptomic mechanisms underlying CIH-exaggerated post-MI remodeling remains unclear. In this study, we used RNA-sequencing to construct the expression profiles of cardiac mRNAs, microRNAs, and long non-coding RNAs (lncRNA) in four groups of C57BL/6J mice (Sham, CIH, MI, MI + CIH) to evaluate how CIH regulates cardiac remodeling after MI. Compared with the other three groups, the MI + CIH group exhibited 345 lncRNAs, 35 microRNAs, and 5,220 differentially expressed mRNAs. Further analysis showed that CIH led to significant changes in Gene Ontology (GO) enrichment and Kyoto Encyclopedia of Genes and Genomes (KEGG) pathway of the differentially expressed mRNAs. Co-expression network analysis identified two core lncRNAs (Mirt1 and AC125351.1) and two core microRNAs (miR-466i-5p and miR-574-5p) during the development of CIH-exaggerated post-MI remodeling, and they were verified by quantitative real-time PCR (qRT-PCR). LncRNA-mRNA correlation analysis further showed that lncRNA Mirt1 was positively correlated with Apbb1ip and Lcp2. In addition, microRNA-mRNA correlation analysis showed that microRNA miR-466i-5p was positively correlated with Snai2, Cdc27, and Ngfr. Furthermore, combining with lncRNA-mRNA and miRNA-mRNA networks, 44 RNAs were identified in the competitive endogenous RNA (ceRNA) network. Mirt1 acts as a ceRNA to bind to miR-466i-5p to further regulate the expression levels of the target gene, thereby aggravating cardiac remodeling after MI. In conclusion, our study provides a systematic perspective on the potential functions of mRNAs, microRNAs, and lncRNAs in CIH-exaggerated post-MI cardiac remodeling. Our data suggest that lncRNA Mirt1 may be the most critical regulator of MI aggravated by CIH.

## Introduction

The incidence of myocardial infarction (MI) has increased rapidly over recent years, and the mortality rate associated with MI has steadily increased in the past 10 years (Yeghiazarians et al., 2021). The mean annual hospitalization expenses for MI have gradually become a serious public health problem. Despite advances in reperfusion therapy and the fact that various measures have been taken to control traditional cardiovascular risk factors (smoking, hypertension, dyslipidemia, and diabetes), the incidence and mortality rate of MI have not been reduced effectively. This suggests that other risk factors may have serious effects on the prognosis of patients with MI.

In recent years, researchers have been increasingly targeting the relationship between obstructive sleep apnea (OSA) and MI. OSA refers to the complete or partial obstruction of the upper airway during sleep that leads to repeated apneas and/or shallow breathing, mainly manifested as sleep fragmentation, repeated snoring, and daytime sleepiness (Javaheri et al., 2017; Cowie et al., 2021). In 2019, global statistics showed that there were approximately 936 million patients with OSA. Of these, 425 million cases were classified as moderate to severe OSA. Moreover, China had the highest number of patients at 176 million (Benjafield et al., 2019). Chronic intermittent hypoxia (CIH) is the main feature of OSA and is known to exaggerate cardiac remodeling after MI (Du et al., 2020). However, the specific contribution of CIH to overall OSA-induced pathological complications, and the transcriptomic mechanisms underlying cardiac remodeling after MI that causes the exaggeration of CIH, remain very unclear.

Long non-coding RNAs (lncRNAs) are RNAs that are longer than 200 nucleotides (nts) and can regulate chromatin modification and the development of diseases by affecting epigenetic programs in the transcriptome (Kaikkonen et al., 2011; St Laurent et al., 2015). Furthermore, lncRNAs can act as endogenous microRNA sponges, thereby participating in mutual competition for the common binding sites of target microRNAs, thus modifying the functions of the target mRNAs to participate in cardioprotection (Zhu et al., 2021). MicroRNAs are a class of small non-coding RNAs, which exert function in the post-transcriptional regulation of gene expression, including cell growth, differentiation, development, and apoptosis (Saliminejad et al., 2019). However, the effect of CIH on the expression profiles of lncRNAs, microRNAs, and mRNAs, in MI mice and whether specific lncRNAs or microRNA are critical for CIH-exaggerated post-MI cardiac remodeling remains to be elucidated.

RNA sequencing is a useful method with which to identify gene function categories, important pathways, molecular interactions, and signal transduction networks and this method can also predict the mechanisms that underlie disease progression. To characterize the dynamic patterns of lncRNA, microRNA, and mRNA expression, and their potential correlation to cardiac remodeling after MI, we used RNA-sequencing to investigate the expression profiles of lncRNA, microRNA, and mRNA between Sham, MI, CIH, and MI + CIH groups of mice. Next, we generated and combined lncRNA-mRNA and microRNA-mRNA networks and identified two lncRNAs and two microRNAs to construct a competing endogenous RNA (ceRNA) network. Node analysis showed that Mirt1 and miR-466i-5p exhibited the largest degrees. These results confirmed that Mirt1 and miR-466i-5p were significantly associated with CIH-exaggerated post-MI cardiac remodeling and maybe the most critical transcriptional regulators in cardiac remodeling after MI.

## Materials and Methods

### Animal Model

Adult male C57BL/6J mice (8–10 weeks old) were randomly assigned to the four experimental groups (Sham, MI, CIH, and MI + CIH groups). In the model groups, MI mice were first anesthetized with 2% isoflurane, and then ligated the left anterior descending artery (LAD) by left thoracotomy to induce surgical MI. Sham-operated control mice (Sham) underwent the same left thoracotomy, except that the LAD was not tied. For CIH, mice were subjected to intermittent hypoxia (5% O_2_ at nadir, 20 cycles/h) for 4 weeks. The MI + CIH mice were exposed to CIH for 4 weeks after MI surgery. At the end of exposure, all of the experimental animals were anesthetized with 2% isoflurane and then killed. Hearts were then collected from each mouse for RNA extraction. All animal handling complied with the standard animal welfare regulations of Capital Medical University. This study was approved by the Institutional Animal Care and Use Committee of Capital Medical University, Beijing, China (2013025).

### Echocardiography

Mice were examined on a Visual Sonics Vevo 2,100 system using a30 MHz-Transducer (MS-400; Visual Sonics). First, the mice were weighed, anesthetized, and fixed on an operating table. The angle of the probe was adjusted to obtain a long axis view of the left ventricle. Then, the probe was rotated 90° clockwise to obtain each view of the short axis of the left ventricle. The value used for analysis was the mean value over the three measured cardiac cycles.

### RNA Preparation, Transcriptome Sequencing, and Analysis

TRIzol reagent (Invitrogen, Carlsbad, Canada) was used to extract total RNA from heart tissue. A NanoDrop 2000microspectrophotometer was used for purification. An Agilent 2,100 Bioanalyzer and an Agilent RNA 6000 Nano Kit were used to assess RNA integrity. Once RNA concentration had been quantified, we used a Small RNA Sample Pre-Kit to build a library from total RNA, use the special structure of small RNAs at the 3′ and 5′ ends (the 5′ end has a complete phosphate group while the 3′end has a hydroxyl group). Next, we added adapters to both ends of the small RNAs and then used reverse transcription to synthesize cDNAs. Polymerase Chain Reaction (PCR) was then used to amplify target DNA fragments, amplicons were then separated by PAGE. Finally, the cDNA library was recovered by cutting out appropriate parts of the gel. Once the cDNA library had been constructed, we used a Qubit2.0 for preliminary quantification. Next, we diluted the cDNA to 1 ng/μl and used an Agilent 2,100 to detect the insert size of the library. Once the insert size had met our expectations, we use a Quantitative PCR (qPCR) method to determine the effective concentration of the library. Quantification was carried out very precisely to ensure that the library was of good quality (an effective library concentration > 2 nM). Following final library quality control, we performed HiSeq/MiSeq Illumina sequencing. A random variance model (RVM) *t*-test was applied to filter differentially expressed genes according to the *p*-value threshold. Hierarchical clustering was then employed to analyze differentially expressed lncRNAs, microRNAs, and mRNAs. The RNA-sequencing (RNA-seq) data has been uploaded into a public database (accession number GSE138008). The scripts used to reproduce the analysis are available from the Bioconductor repository (http://www.bioconductor.org/packages/release/bioc/html/DESeq.html).

### Quantitative Real-Time PCR

Quantitative real-time PCR (qRT-PCR) was performed to validate the expression of significantly altered mRNAs, microRNAs, and/or lncRNAs, in heart tissues from the mouse model. Total RNAs were extracted using TRIzol reagent (Invitrogen, Carlsbad, Canada). First-stand cDNA was synthesized using a First Strand cDNA Synthesis kit (Thermo Fisher Scientific). qRT-PCR was performed with a SYBR Green Master Mix (Takara) to determine the relative mRNA levels of the indicated genes. 18s served as an endogenous control so that we could normalize the expression levels of each target gene. The 2−ΔΔCt value was calculated to indicate the relative expression levels of each gene. Sequences of the qRT-PCR primers are shown below:

mouse 18s: (5′-GCG​GCG​GAA​AAT​AGC​CTT​TG-3′ forward; 5′GAT​CAC​ACG​TTC​CAC​CTC​ATC-3′ reverse); mouse AC152979.5: (5′- GGG​CAG​CCT​ACA​CAG​CTA​CA-3′ forward; 5′-CAC​GCT​CAC​TGT​CTC​ACC​CA-3′ reverse); mouse Gm45055: (5′- CCT​GCT​CTA​CAG​TGC​GAC​AAC​ATG-3′ forward; 5′-TCT​CAG​CTT​CAG​GAC​CAG​CGA​TAG-3′ reverse); mouse Mirt1: (5′-GCA​CCA​TCT​CAG​TGA​CAG​CTT​CC-3′ forward; 5′-CAG​TTG​GCT​TGG​AGG​AGG​ACA​ATC-3′ reverse); mouse AC125351.1: (5′- CAA​GAA​CCT​GGC​GAC​TTC​ACC​TG-3′ forward; 5′- TGC​CTC​AGC​CTC​CTG​TGT​AGC-3′ reverse); mouse mmu-miR-466i-5p: (5′- cgT​GTG​TGT​GTG​TGT​GTG​TGT​G-3′ forward); mouse mmu-miR-574-5p: (5′- cgT​GAG​TGT​GTG​TGT​GTG​AGT​GTG​T-3′ forward).

### Bioinformatics Analysis

Next, we attempted to identify significant functions and pathways related to the differentially expressed genes. For this, we applied Gene Ontology (GO) and Kyoto Encyclopedia of Genes and Genomes (KEGG) pathway analysis. GO analysis usually divides corresponding genes by three aspects: biological process (BP), molecular function (MF), and cellular component (CC). KEGG pathway analysis was used to identify significant pathways related to the differential genes according to the KEGG database. Two-sided Fisher’s exact tests and Chi-squared tests were used to analyze and compare the significantly different functions and pathways. We also used false discovery rate (FDR) and corrected *p* values < .05 to identify important expression profiles.

Next, we performed co-expression network (lncRNA-mRNA and mRNA-microRNA) analysis to investigate functional annotation. The expression correlation network was built on the normalized signal intensity of differentially expressed mRNAs and lncRNAs/microRNAs. The R function cor. test (Hmisc and corrplot) was used to compute the Pearson’s correlation coefficient for mRNA-lncRNA/microRNA pairs. Significant correlation pairs we identified using a correlation cutoff of 0.99. Pearson’s correlation was used to select significant lncRNA-mRNA or mRNA-microRNA correlation pairs. The network was visualized by Cytoscape (version: 3.6.0) software. The degree was calculated to measure a gene or lncRNA centrality within a network.

A lncRNA/microRNA target pathway network was built according to the relationships created by significant pathways and lncRNAs/microRNAs, as well as the relationships among lncRNAs/microRNAs and pathways. In the target pathway network, a round node represents a pathway, a square represents a lncRNA/microRNA, and relationships between them are represented by edges. The degree of each lncRNA/microRNA was the number of pathways regulated by that lncRNA/microRNA, and the degree for each pathway was the number of lncRNAs/microRNAs that regulated the pathway. The size of the node represents the strength of the correlation. Core lncRNAs/microRNAs and pathways in the network had the biggest degrees.

A ceRNA network was constructed to identify ceRNA mechanisms based on differentially expressed lncRNAs, miRNAs, and mRNAs. RNA transcripts can combine with miRNAs by the miRNA response element (MRE). Therefore, we could identify competitive relationships between RNA transcripts in the process of combining MREs by predicting MREs and computing free energy. First, miRNA-mRNA and miRNA-lncRNA target relationships were predicted by a target prediction database. Pearson’s correlation coefficient (PCC) was then computed between matched lncRNA-mRNAs based on their expression data. Then, the PCC between miRNA-mRNA and miRNA-lncRNA was computed. For a given lncRNA-mRNA pair, both mRNA and lncRNA were targeted by a common miRNA and co-expressed negatively with this miRNA. Finally, the miRNA-mRNA-lncRNA network was identified as competing triplets. The network was visualized by Cytoscape software (version: 3.6.0). In the ceRNA network, square nodes represent lncRNAs, triangle nodes represent microRNAs, and rhombi nodes represent mRNAs. The lines between nodes indicate a correlation. The size of a node represents the strength of the correlation.

### Statistical Analysis

Normally distributed data were compared using the independent-sample *t*-tests (two groups) or one-way analysis of variance (ANOVA) with Bonferroni’s procedure for multiple comparison tests (three groups). Non-normally distributed and unpaired data were compared using the Mann-Whitney U test or the Kruskal-Wallis test. GraphPad Prism version 6.0 (GraphPad Software Inc., San Diego, CA) and SPSS version 24.0 (SPSS Inc., Chicago, IL) were used for statistical analyses. *p* < .05 was considered significant.

## Results

### CIH Exaggerated Post-MI Cardiac Remodeling and Reduced Survival

**FIGURE 1:**
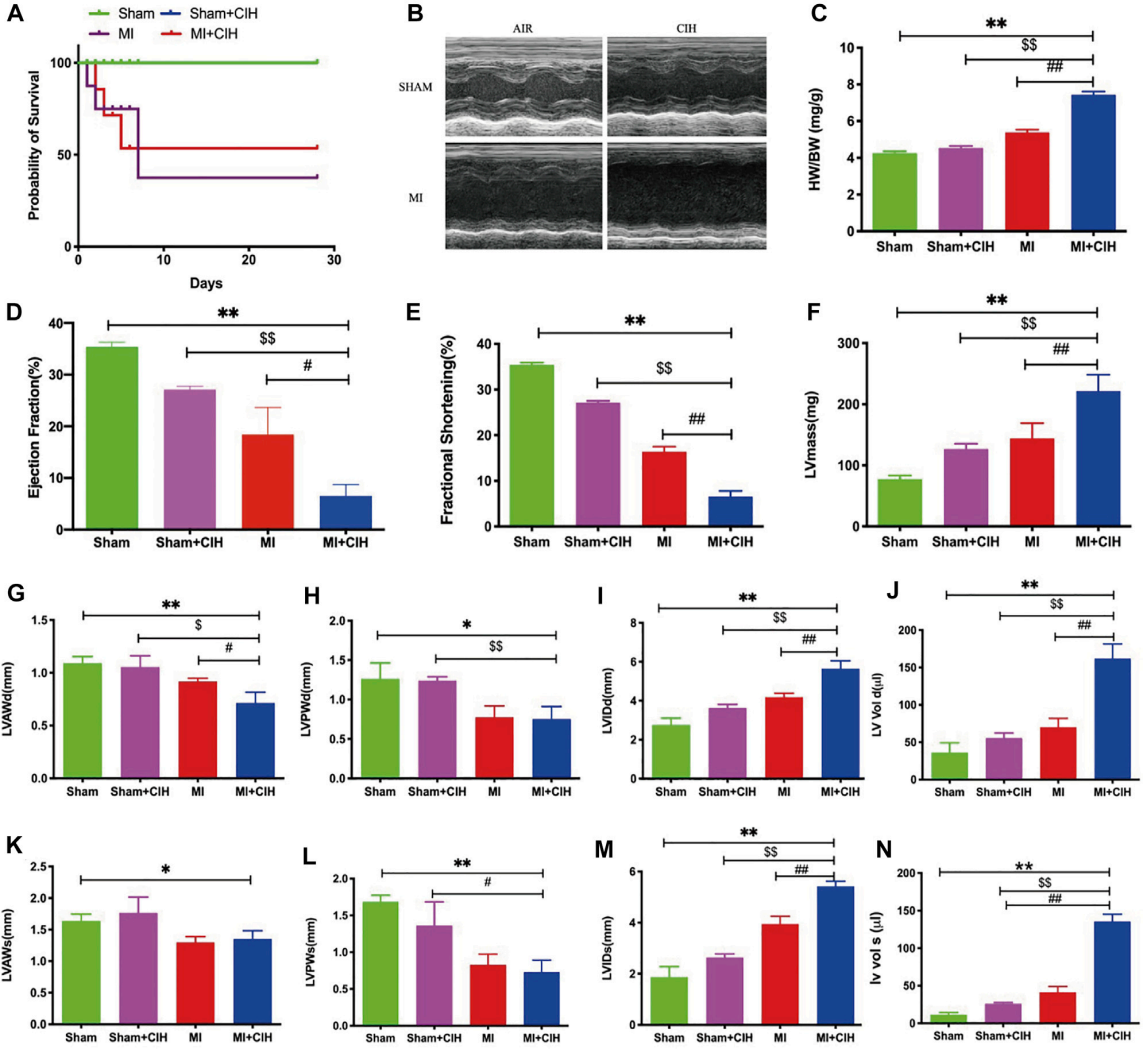
CIH increased post-MI cardiac injury and reduced survival. The effect of CIH upon survival and LV function was determined 28 days after MI. WT mice were subjected to MI or MI with CIH operation. **(A)** Survival curve (*p* = .0275). **(B)** Cardiac function was examined by echocardiography, and four representative sets of left ventricular M-mode echocardiography images were displayed. **(C)** Statistics of HW/BW. **(D–N)** Quantification of EF, FS, LVmass, LVAWd, LVAWs, LVPWd, LVPWs, LVIDd, LVIDs, LV Vol-d, and LV Vol-s, from the echocardiographic analysis. *n* = 6–8/group, SHAM vs. MI + CIH: *p < .05, **p < .01; SHAM vs. MI + CIH: $*p* < .05, $$*p* < .01; MI vs. MI + CIH: #*p* < .05, ##*p* < .01. Abbreviations: HW, heart weight; BW, body weight; EF, ejection fraction; FS, fractional shortening; LVmass, Left Ventricular Mass; LVAWd, Left Ventricular Diastolic Anterior Wall; LVAWs, Left Ventricular Systolic Anterior Wall; LVPWd, Left Ventricular Diastolic Posterior Wall; LVPWs, Left Ventricular Systolic Posterior Wall; LVIDd, Left Ventricular Internal Diastolic Diameter, LVIDs, Left Ventricular Internal Systolic Diameter; LV Vol-d, Left Ventricular Diastolic Volume; LV Vol-s, Left Ventricular Systolic Volume.

CIH is the main feature of OSA-related cardiovascular diseases and is characterized by repeated circulating hypoxemia and re-oxidation. In order to determine the effect of CIH on cardiac remodeling after infarction, we exposed mice undergoing left coronary artery ligation to a three-gas animal incubator to simulate OSA conditions. Compared to the other three groups, the survival rate was significantly decreased in the MI + CIH group (*p* = .0275) (Figure 1A). To determine whether CIH exposure may differentially affect post-MI pathological remodeling, we determined heart weight (HW) and analyzed cardiac function by echocardiography (Figure 1B). MI mice subjected to CIH exhibited a high cardiac hypertrophy index, as indicated by HW/body weight (BW) ratios at 28 days (Figure 1C). MI mice subjected to CIH showed markedly exacerbated post-MI remodeling and heart failure (HF). As shown in Figures 1D–N, MI with CIH induced cardiac dysfunction, as evidenced by the reduction of ejection fraction (EF), fractional shortening (FS), left ventricular systolic anterior wall (LVAW), and left ventricular posterior wall (LVPW), within 2 weeks after MI. Meanwhile, Left Ventricular Mass (LVmass), Left Ventricular Internal Diameter (LVID), and Left Ventricular Volume (LV VOL) were significantly increased (*p* < .05). Collectively, these data indicate that CIH exposure in MI is an important factor that can increase pathological remodeling.

### Hierarchical Clustering Demonstrated How Differentially Expressed mRNAs, microRNAs and lncRNAs Were Related

**FIGURE 2:**
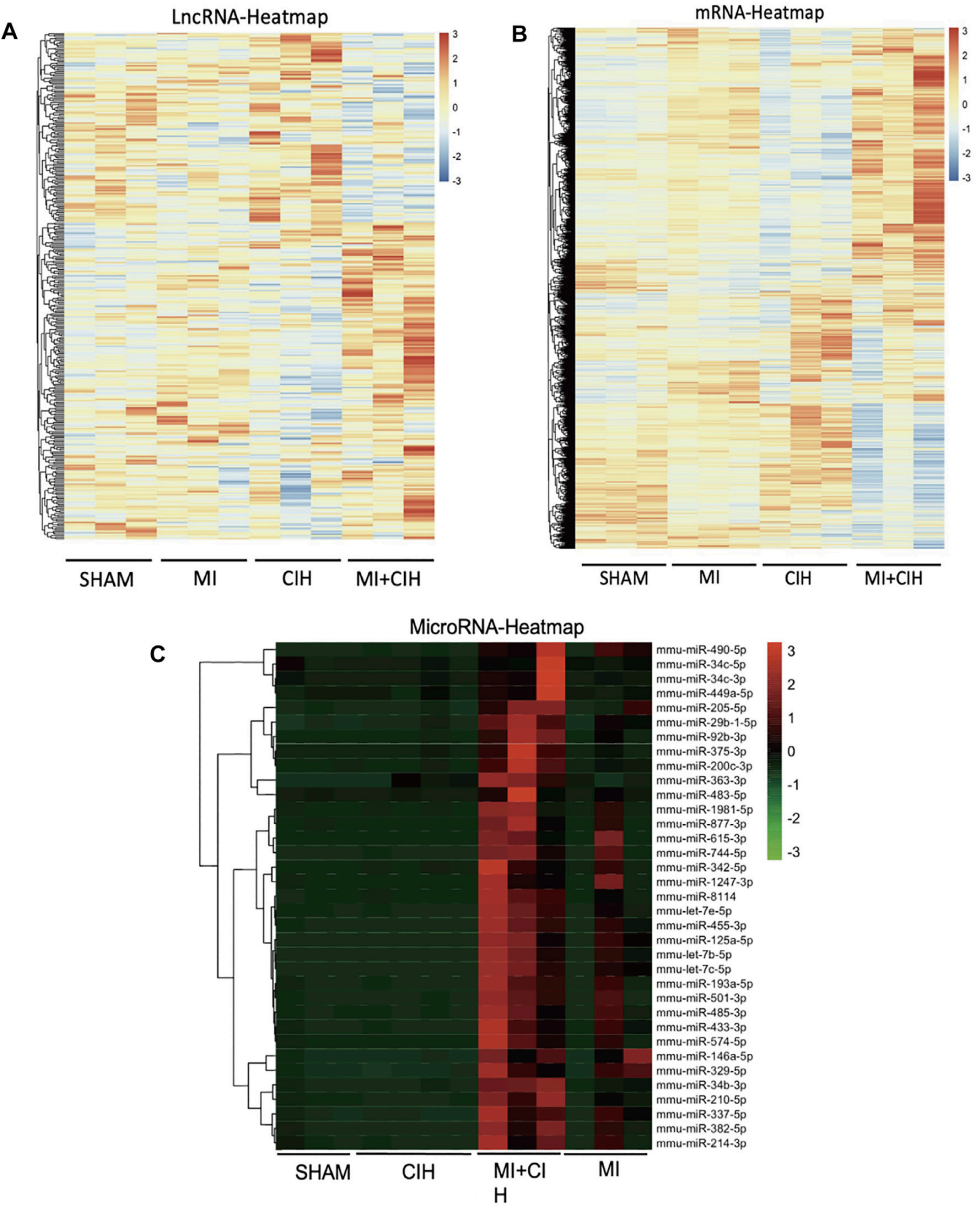
Hierarchical cluster shows the relatedness of differentially expressed genes. **(A)** Heat map of the relative abundance of significantly changed LncRNAs by Sham, MI, CIH, and MI + CIH groups. **(B)** Heat map of the relative abundance of significantly changed mRNA by Sham, MI, CIH, and MI + CIH groups. **(C)** Heat map of the relative abundance of significantly changed microRNAs by Sham, MI, CIH, and MI + CIH groups. *n* = 8/group.

Next, we investigated the transcriptomic mechanisms associated with CIH-exaggerated post-MI cardiac remodeling and assessed how CIH modulates the expression profiles of mRNAs, microRNAs, and/or lncRNAs during MI. To do this, we performed transcriptome-sequencing analysis on each group of mice and identified the expression profiles of mRNAs, microRNAs, and lncRNAs in mouse heart tissue. As a result, a total of 5,220 differentially expressed genes (fold change ≥5, *p*-value < .05), 35 differentially expressed microRNAs (fold change ≥30, *p*-value < .05), and 345 differentially expressed lncRNAs (fold change ≥1.5, *p*-value < .05) were identified in the MI + CIH group when compared with the sham group. The relative abundances of the mRNAs, microRNAs, and lncRNAs, affected by CIH under MI injury are shown in Figure 2. Clearly, these differentially expressed mRNAs, microRNAs, and lncRNAs showed different expression patterns among the groups.

### GO and KEGG Analyses Showed That CIH Induced Significant Changes in mRNAs and microRNAs

**FIGURE 3:**
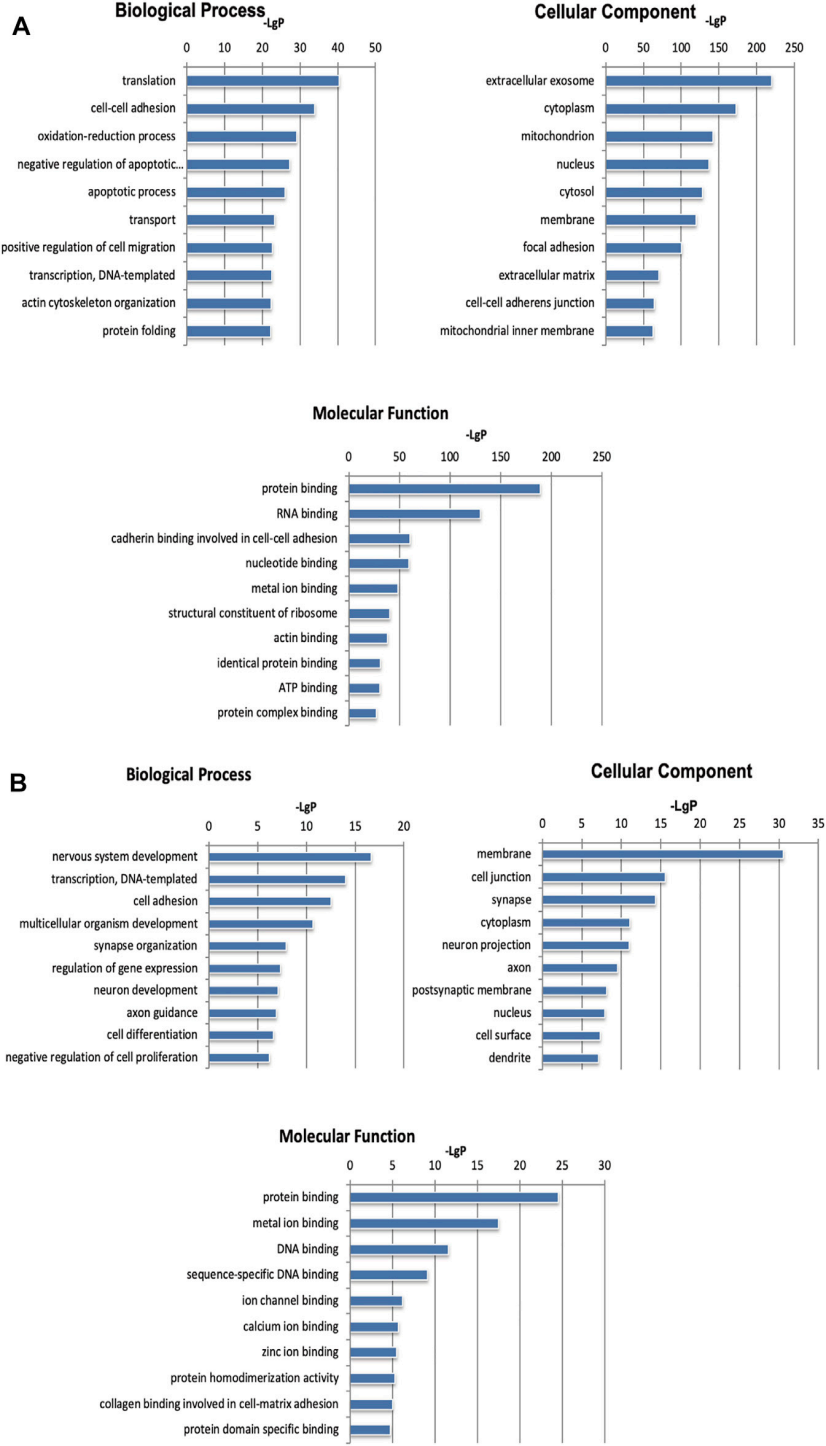
GO analysis of differentially expressed mRNAs and microRNAs induced by CIH. **(A)** GO enrichment analysis for mRNAs. **(B)** GO enrichment analysis for microRNAs. The abscissa is -LgP. The bigger the -LgP, the smaller the *p*-value.

**FIGURE 4:**
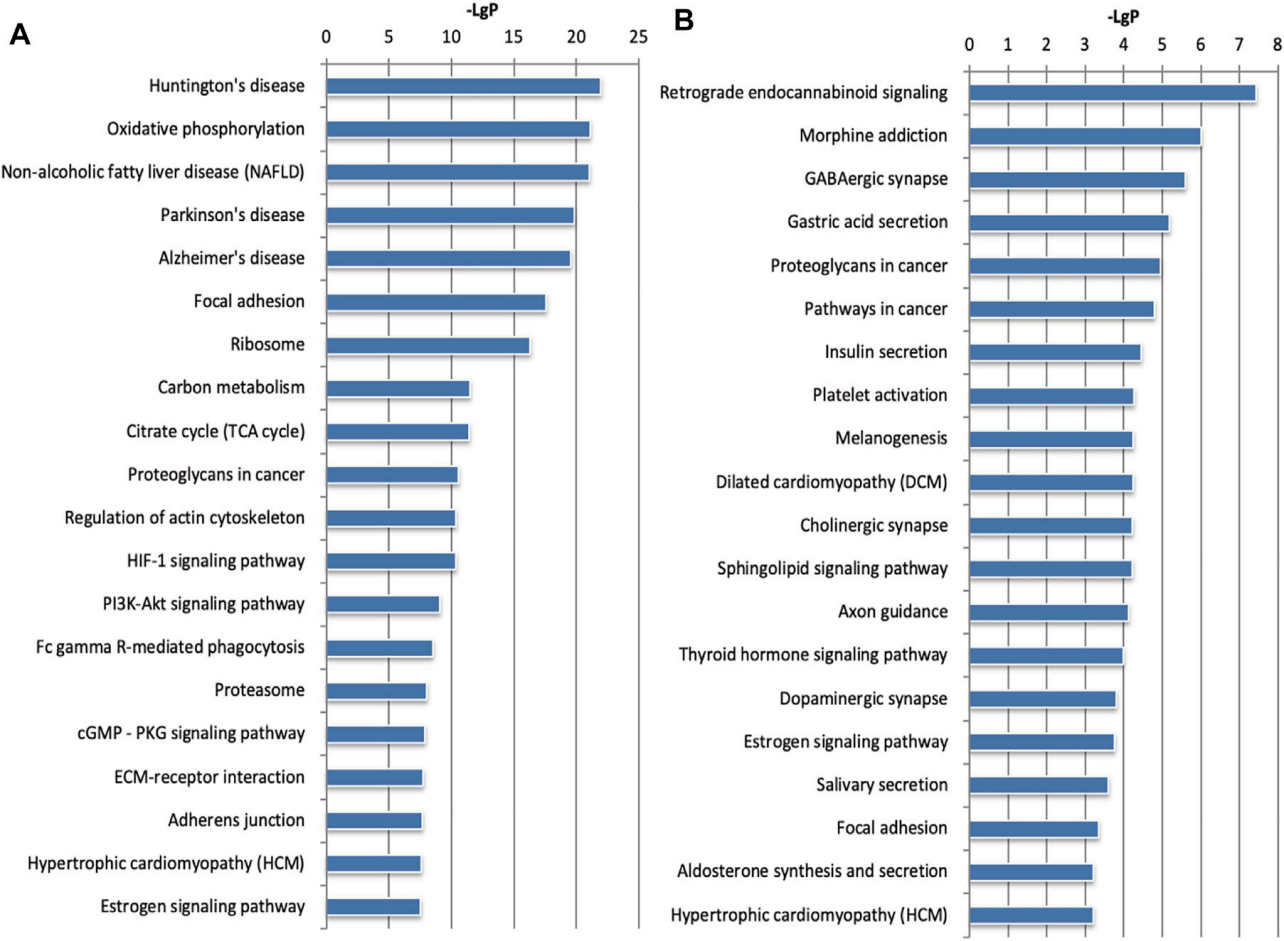
KEGG pathway analysis for the mRNAs and microRNAs regulated by CIH. **(A)** KEGG pathway enrichment analysis for mRNAs. **(B)** KEGG pathway enrichment analysis for microRNAs. The abscissa is -LgP. The bigger the -LgP, the smaller the *p*-value.

To further study the biological functions of differentially expressed genes, we performed mRNA and microRNA enrichment analysis (FDR <0.05) based on a dataset of biological processes, cell components, and molecular functions, created by the GO database. We found that differentially expressed mRNAs were enriched in terms of translation, cell-cell adhesion, and oxidation-reduction processes (for biological processes), and extracellular exosomes, cytoplasm, and mitochondria (for cellular components). For molecular function, the differentially expressed mRNAs were mainly enriched in terms of protein binding, RNA binding, and cadherin binding (Figure 3A). Next, enrichment analysis was carried out for the microRNAs, we found that differentially expressed microRNAs were enriched in transcription, DNA-templates, and cell adhesion (for biological processes); membranes, cell junctions, and synapses (for cellular components); and protein binding, metal ion binding, and DNA binding (for molecular function) (Figure 3B). Based on the KEGG database, the most enriched pathways corresponding to the differentially expressed mRNAs were associated with oxidative phosphorylation and carbon metabolism (Figure 4A). Next, we performed pathway enrichment analysis using the CIH-associated microRNAs. As shown in Figure 4B, several signaling pathways were activated, including GABAergic synapses, the sphingolipid signaling pathway, and the thyroid hormone signaling pathway.

### Generation of a lncRNA-mRNA Co-Expression Network and Target Pathway

**FIGURE 5:**
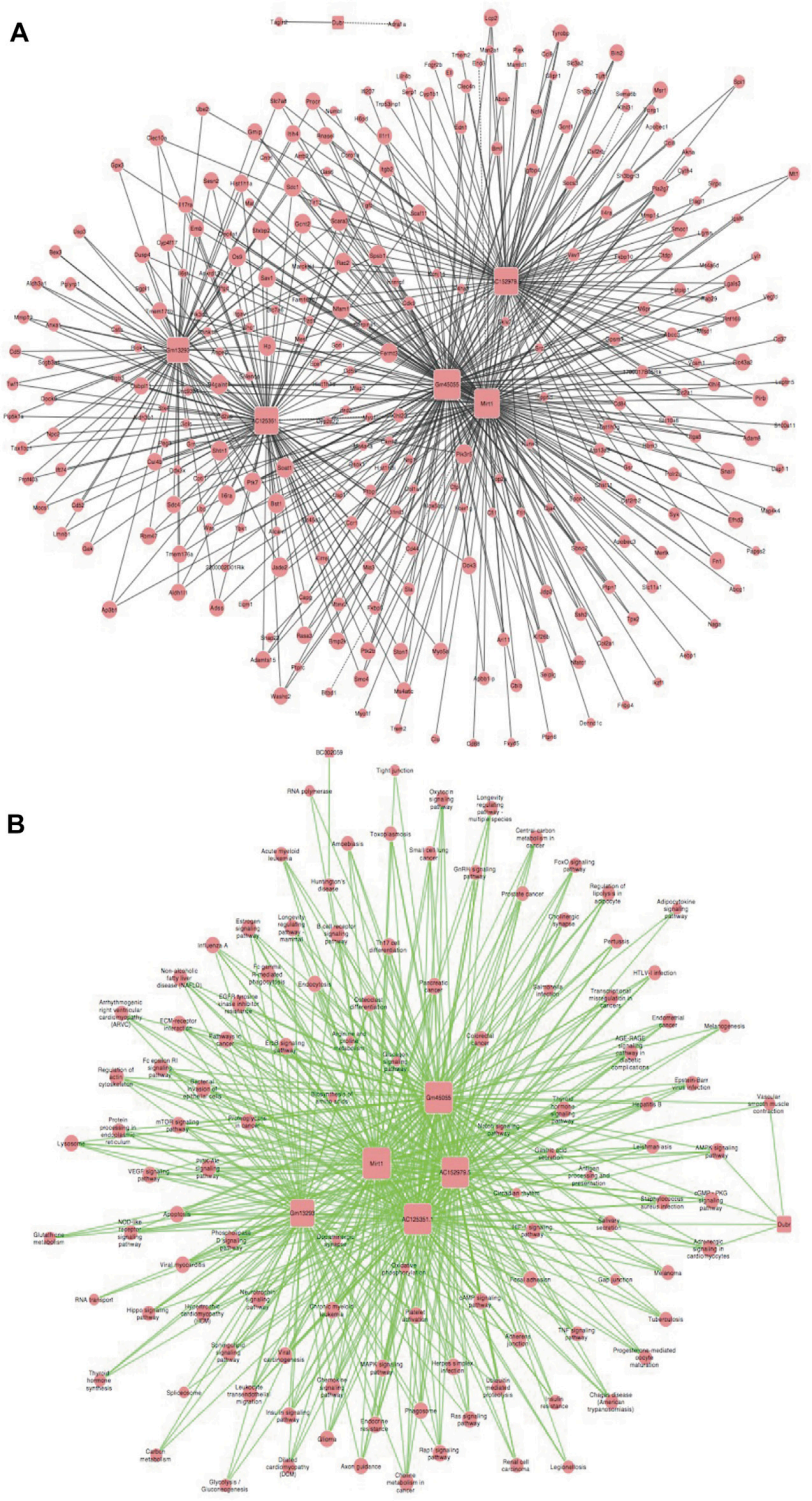
LncRNA-mRNA co-expression correlation network and target pathway network of core lncRNAs. **(A)** LncRNA-mRNA expression correlation network analysis of core lncRNAs (Gm45055, Mirt1, AC152979.5, and AC125351.1) and their correlated mRNAs regulated by CIH post-MI. Square nodes and round nodes represent lncRNAs and mRNAs. The lines between nodes indicate a correlation, with a solid line representing positive correlation and a dotted line representing a negative correlation. The size of node represents the correlation strength. **(B)** Network of the core lncRNAs (Gm45055, Mirt1, AC152979.5, and AC125351.1) and target pathways. Square nodes represent lncRNAs, round nodes represent pathways, and relationships between them are represented by edges. The size of node represents the correlation strength.

We established a co-expression network for lncRNA and mRNA based on microarray results and used this data to identify the possible linkage between lncRNAs and downstream mRNAs in MI + CIH mouse heart tissue. LncRNA-mRNA co-expression Network was built according to the relationships of significant mRNA and lncRNAs, as well as the relationships among lncRNAs and mRNA. Using the methods of graph theory, we evaluated the regulatory status of lncRNAs and mRNA; the evaluation criteria were the degrees of lncRNAs and mRNA in the network. The degree of each lncRNA was the number of mRNAs regulated by that lncRNA, and the degree of each mRNA was the number of lncRNAs that regulated the mRNA. Key lncRNAs and mRNA in the network had the biggest degrees. Four significant lncRNAs (with a degree >80) were identified in the network map (Figure 5A). Therefore, we think these four core lncRNAs are of great importance in the four groups of C57BL/6J mice (Sham, CIH, MI, MI + CIH). We selected these four for subsequent analysis and verification. A total of four lncRNAs and 815 mRNAs were selected to generate a network map. The nodes that showed the highest degrees included Gm45055 (degree = 135), Mirt1 (degree = 117), AC152979.5 (degree = 98), and AC125351.1 (degree = 96). For the core lncRNAs (Gm45055, Mirt1, AC152979.5, and AC125351.1), we observed that lncRNA Gm45055 was positively linked with Gcnt2 and Dok3, and that lncRNA Mirt1 was positively correlated with Apbb1ip and Lcp2. LncRNA AC152979.5 was positively correlated with Socs3 and Tgfbi. LncRNA AC125351.1 was positively correlated with Osbpl11 and Emb. The core lncRNAs were indirectly connected by their corresponding mRNAs. These mRNAs and lncRNAs were enriched and clustered for further analysis.

Based on the functions and interactions of these differentially expressed lncRNAs, we next identified significant lncRNA target pathway networks (Figure 5B). All of the core lncRNAs [Mirt1 (degree = 98), Gm45055 (degree = 97), AC125351.1 (degree = 94), and AC152979.5 (degree = 92)], exhibited interaction networks with the HIF-1 and PI3K-Akt signaling pathways. The HIF-1 signaling pathway, the PI3K-Akt signaling pathway, and the core lncRNAs, therefore represent putative players that are most likely to contribute to post-MI cardiac remodeling under CIH conditions.

### Generation of a Co-Expression Network (microRNA-mRNA) and microRNA Target Pathway Network

**FIGURE 6:**
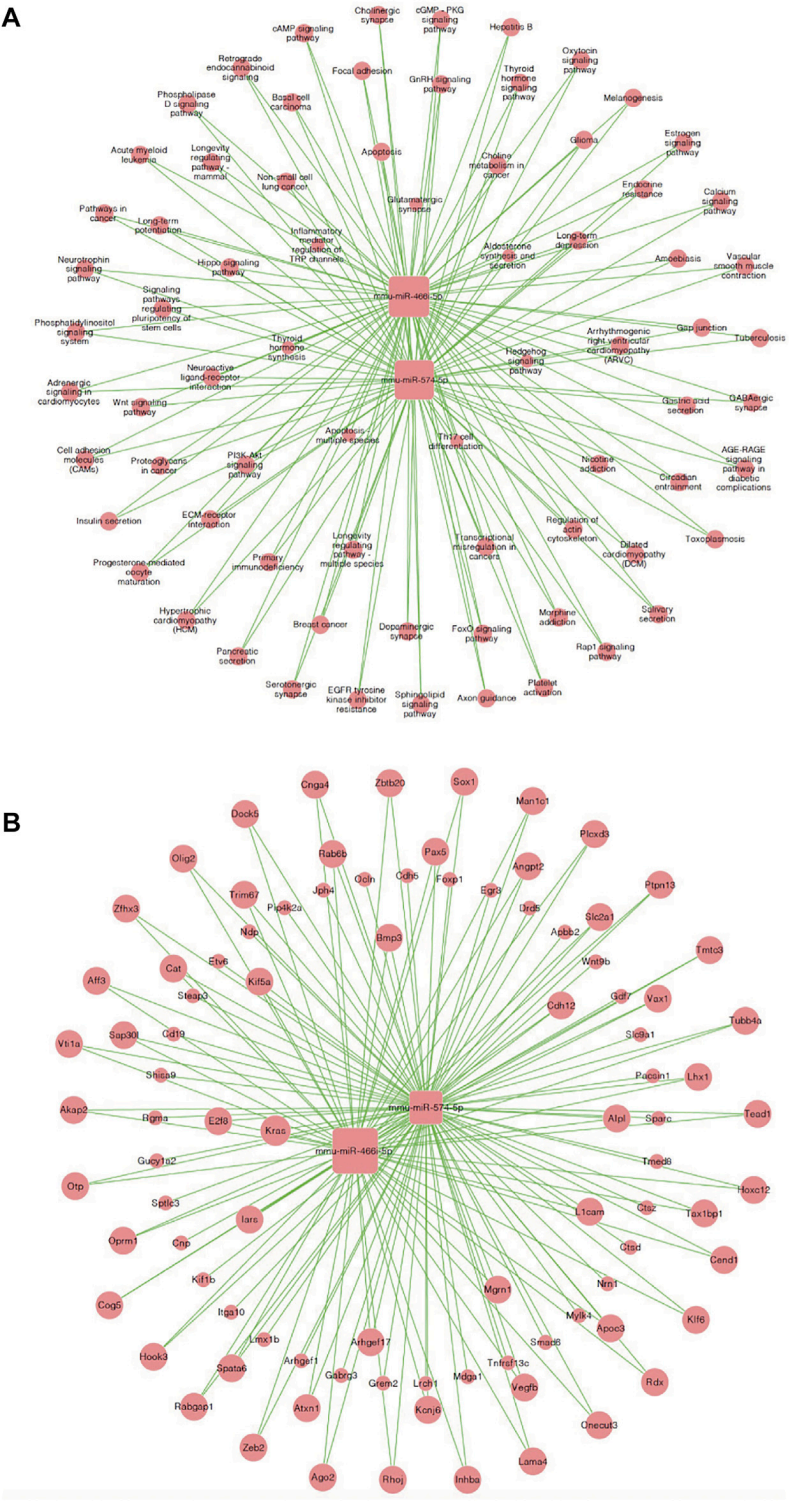
MicroRNA-mRNA co-expression correlation network and target pathway network of core MicroRNAs. **(A)** MicroRNA—mRNA expression correlation network analysis of core MicroRNAs (miR-466i-5p and miR-574-5p) and their correlated mRNAs regulated by CIH post-MI. Square nodes and round nodes represent MicroRNAs and mRNAs. The lines between nodes indicate a correlation. The size of node represents the correlation strength. **(B)** Network of the core MicroRNAs (miR-466i-5p and miR-574-5p) and target pathways. Square nodes represent MicroRNAs, round nodes represent pathways, and relationships between them are represented by edges. The size of node represents the correlation strength.

Similarly, we established a microRNA-mRNA co-expression network based on our RNA-sequencing results to predict possible associations between microRNAs and target genes in MI + CIH mouse heart tissue (Figure 6A). We identified microRNA miR-466i-5p (degree = 466) and miR-574-5p (degree = 92) during the development of MI-induced cardiac remodeling and exposure to CIH. When considering these core microRNAs (miR-466i-5p and miR-574-5p), we observed that miR-466i-5p was positively correlated with 932 mRNAs, including Snai2, Cdc27, and Ngfr. MicroRNA miR-574-5p was positively correlated with 184 mRNAs, including Tmed8 and Ctsz. Next, we identified an important microRNA target pathway network based on the KEGG network. As shown in Figure 6B, miR-466i-5p and miR-574-5p were associated with the PI3K-Akt signaling pathway, the Wnt signaling pathway, and the cAMP signaling pathway.

### Verification of Key Genes by Real-Time PCR Analysis

**FIGURE 7:**
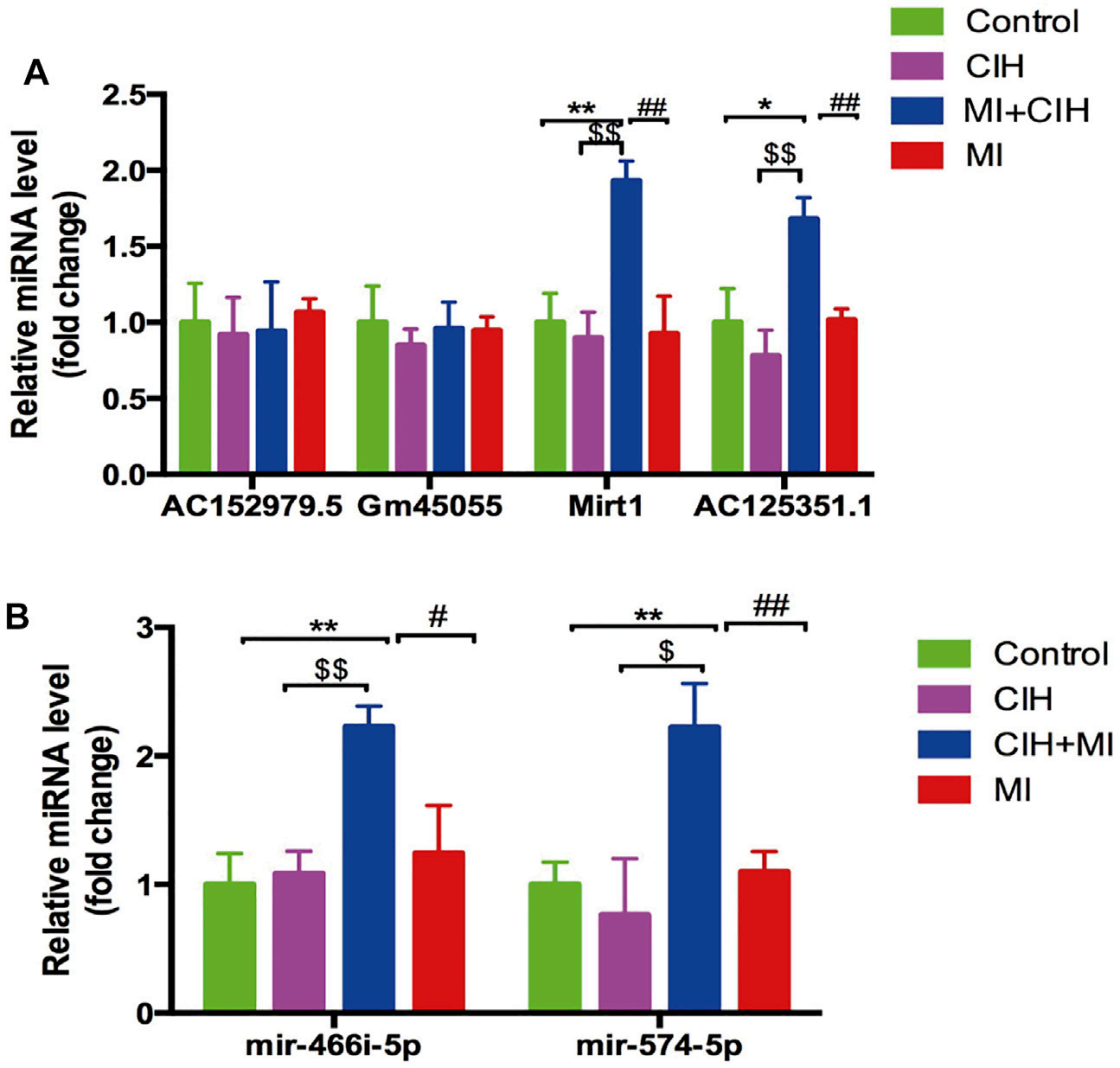
Quantitative real-time PCR (qRT-PCR) validation of four selected lncRNAs and two microRNAs in RNA-sequencing results. **(A)** The expression level of lncRNAs Gm45055, Mirt1, AC152979.5 and AC125351.1. **(B)** The expression level of microRNAs miR-466i-5p and miR-574-5p. SHAM vs. MI + CIH: *p < .05, *p* < .01; SHAM vs. MI + CIH: $*p* < .05, $$*p* < .01; MI vs. MI + CIH: #*p* < .05, ##*p* < .01.

To validate the reliability of the RNA-sequencing results, the four lncRNAs and two microRNAs were selected and validated by qRT-PCR. PCR data showed that the four lncRNAs (Gm45055, Mirt1, AC152979.5, and AC125351.1) and the two microRNAs (miR-466i-5p and miR-574-5p) exhibited the same trends in expression changes and the same significant differences when compared between RNA-sequencing and RT-qPCR analyses (Figure 7). Consequently, these may represent key target genes in CIH-exaggerated post-MI cardiac remodeling. The key genes associated with CIH-exaggerated post-MI cardiac remodeling are shown in Table 1.

**TABLE 1:** The detailed information of top genes ranked by degree after analysis of LncRNA/microRNA -mRNA co-expression correlation network analysis.

Gene name	Biotype	Position	Degree	Fold change	FDR
Gm45055	lncRNA	chr7:64001885-64020959: +	135	1.266850501	0.01055173
Mirt1	lncRNA	chr19:53441212-53464796: −	117	1.289953773	0.01636217
AC152979.5	lncRNA	chr10:51526340-51531161: +	98	1.618291764	0.03795368
AC125351.1	lncRNA	chr12:81717830-81720052: −	96	1.453047958	0.00305501
mmu-miR-466i-5p	microRNA	—	466	54.68407947	0.01690546
mmu-miR-574-5p	microRNA	—	92	43.61002412	1.7116E-05

FDR, false discovery rate.

### CeRNA Analyses Identified Potential lncRNA/miRNA/mRNA Interactions

**FIGURE 8:**
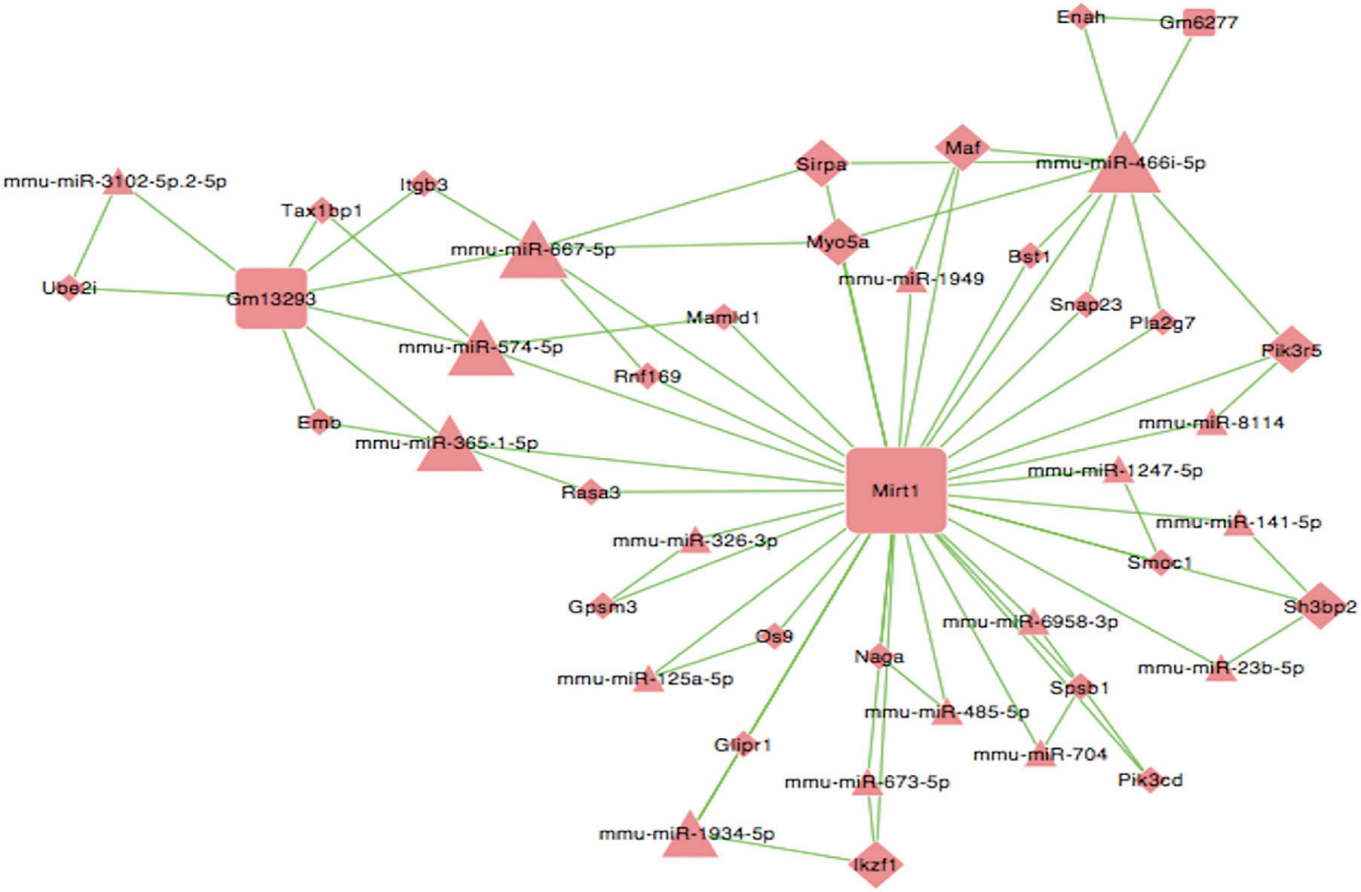
LncRNA–miRNA–mRNA ceRNA network. Square nodes represent lncRNAs, triangle nodes represent microRNAs, and rhombus nodes represent mRNAs. The lines between nodes indicate a correlation. The size of node represents the correlation strength.

Next, we constructed a competing endogenous RNA (ceRNA) network and used this to identify ceRNA mechanisms based on differentially expressed lncRNAs, miRNAs, and mRNAs. Some lncRNAs act as ceRNAs that participate in mutual competition for the common binding sites of target miRNAs and thus modify the functions of target mRNAs. To further investigate the significant functions of differentially expressed genes, we constructed a lncRNA-miRNA-mRNA ceRNA network. There were 44 nodes (including three lncRNAs, 17 miRNAs, and 24 mRNAs) in the ceRNA network (Figure 8). A key lncRNA, Mirt1 (degree = 35), was identified in our analysis and it was connected with 25 miRNAs, including miR-466i-5p (degree = 10), miR-667-5p (degree = 6), and miR-365-1-5p (degree = 4). The network hub features of Mirt1 suggest that thus lncRNA can function as a sponge for miR-466i-5p, thus further regulating the level of target genes such as Enah and Maf. Mirt1 and miR-466i-5p may be the most critical transcriptional regulators in CIH-exaggerated post-MI cardiac remodeling.

## Discussion

In the present study, we used RNA sequencing to analyze the expression profiles of mRNAs, microRNAs, and/or lncRNAs in the myocardial tissue of four groups of C57BL/6J mice (Sham, CIH, MI, MI + CIH). Compared with other groups, the animals in the MI + CIH group were associated with 345 lncRNAs, 35 microRNAs, and 5,220 differentially expressed mRNAs. In addition, ceRNA analysis suggested that microRNA miR-466i-5p and lncRNA Mirt1 may be the most critical transcriptional regulators in the cardiac remodeling that occurs after MI.

OSA promotes cardiac remodeling after MI, identifying targets for early prevention or intervention in this process is of great significance if we are to delay the deterioration of heart function and improve the prognosis of patients. CIH is a process involving long-term exposure to repeated episodes of hypoxia followed by reoxygenation, which is considered to be a prominent feature of OSA pathophysiology (Semenza and Prabhakar, 2007). However, the effect of CIH on the expression profiles of lncRNAs, microRNAs, and mRNAs in MI mice, and whether specific lncRNAs or microRNA are critical for CIH-exaggerated post-MI cardiac remodeling, remains unclear. RNA sequencing is a new generation of sequencing technology that could expand our understanding of the molecular pathogenesis of CIH and how this regulates cardiac remodeling after MI (Kowara et al., 2021).

Cardiac remodeling is the main pathophysiological process after MI, which can lead to ventricular dilatation and cardiac insufficiency, and is a key factor affecting the prognosis of patients (Spinale, 2007). A number of clinical studies have shown that OSA is closely associated with pathological cardiac remodeling after MI (Dumitrascu et al., 2013; Du et al., 2020; Yeghiazarians et al., 2021). Cardiac remodeling involves many cellular and molecular pathways, and its pathophysiological processes involved mainly include interstitial fibrosis, cardiomyocyte hypertrophy, and the death of cardiomyocytes (such as necrosis, apoptosis, or autophagy). In this study, we confirmed the contribution of CIH to MI mice. The morphological changes of CIH 4 weeks after infarction have been previously reported to be severe, including an enlarged heart, infarct size, and cardiac dysfunction (reductions in LVEF, FS, and LVAW). These observations are consistent with our findings.

RNA-sequencing has shown that many lncRNAs exhibit strong regulatory effects on pathological cardiac remodeling (Hao et al., 2019; Zhou et al., 2019). Therefore, the manipulation of lncRNA expression levels, by inhibiting up-regulated lncRNAs after MI or by increasing down-regulated lncRNAs, maybe a new therapeutic strategy with which to inhibit cardiac remodeling after MI (Chen et al., 2021; Han and Yang, 2021; Kowara et al., 2021). In this study, A total of 345 differentially expressed lncRNAs were identified following 4 weeks of CIH after MI. We also identified a number of novel lncRNAs, whose functions of these lncRNAs have yet to be elucidated. We focused on four lncRNAs (Mirt1, Gm45055, AC125351.1, and AC152979.5) and found that all four exhibited an interaction network with the HIF-1 and PI3K-Akt signaling pathways. HIF-*α* signaling contributes to the cardiac remodeling process and can exert significant effects on heart function (Abe et al., 2017). The PI3K-Akt signaling pathway can be activated during MI and myocardial reperfusion to produce cardioprotection-related effects (Madonna et al., 2013). We speculate that the response of key lncRNAs to CIH exaggerated cardiac remodeling after MI may be exerted action *via* the regulation of certain signal pathways (e.g., HIF-1 and PI3K-Akt) to inhibit the expression of corresponding mRNAs, thereby exacerbating the consequences of MI. Compared with lncRNA, microRNA is a relatively conservative endogenous non-coding RNA that is approximately 22 nts in length. microRNAs are known to regulate more than 30% of genes by inhibiting or promoting mRNA translation and play an important role in regulating the biological activity of cells (Huang, 2018). Our results identified 35 differentially expressed microRNAs after 4 weeks of CIH exposure. We identified two core microRNAs (miR-466i-5p and miR-574-5p) in the microRNA-mRNA co-expression network that predicted the association between microRNA and target genes in MI + CIH heart tissue, and these were also associated with the PI3K-Akt signaling pathway.

Indeed, several studies have shown that lncRNAs can act as ceRNAs that sequester miRNAs, thus allowing for de-repression of downstream miRNA targets (Wang et al., 2019; Kang et al., 2021). Such integration between coding and non-coding RNA forms complex ceRNA networks that when dysregulated lead to cardiac remodeling after MI(Wang et al., 2021). We constructed a lncRNA-miRNA-mRNA ceRNA network and found that Mirt1 exhibited an obvious network hub feature, and the highest-degree microRNA related to Mirt1 was miR-466i-5p. Mirt1 is a newly discovered and highly conserved lncRNA that is involved in various pathological processes, including MI and diabetic cardiomyopathy (Vausort et al., 2014; Boon et al., 2016; Zhou et al., 2019). Early RNA-sequencing analysis showed that the expression of Mirt1 in MI tissue showed the most significant increase of all differentially expressed genes. The expression levels of Mirt1 are related to the expression of various pro-fibrotic genes, such as TGF-β and MMP9. The increase of Mirt1 reduces the left ventricular ejection fraction of mice while knocking down the expression of Mirt1 inhibits collagen production and interstitial fibrosis (Zangrando et al., 2014; Liu et al., 2019). Our research also suggested that Mirt1 may play a key role in OSA to aggravate cardiac remodeling after MI. Studies by Saravanan et al. showed that miR-466i-5p can inhibit lipopolysaccharide-induced hepatocyte inflammatory response (Saravanan et al., 2015). In diet-induced obese mice, the inhibition of miR-466s can regulate the anti-inflammatory differentiation of macrophages. Therefore, we speculate that Mirt1 may act as a ceRNA to bind to miR-466i-5p to further regulate the level of target genes, such as promoting fibrosis and inhibiting inflammation, thereby aggravating cardiac remodeling after MI.

Based on the above transcription analysis, we speculate that Mirt1 can function as a ceRNA for miR-466i-5p to modulate the HIF-1 and PI3K-Akt signaling pathways by further targeting the level of corresponding mRNAs. However, to clarify that Mirt1 is indeed the most critical transcriptional regulator in the process of cardiac remodeling after MI, further *in vivo* and *in vitro* experiments are needed to determine the effect of gain- and loss-of-function of Mirt1 on CIH-exaggerated post-MI cardiac remodeling. However, since the CIH and ischemia-reperfusion (IR) models are both hypoxia and reoxygenation models under different conditions, it is difficult for us to simultaneously simulate the injury of CIH combined with IR *in vitro*. In addition, if we want to fully simulate the *in vivo* situation, we need to isolate C57BL/6 adult mouse primary cardiomyocytes. The technology of isolating C57BL/6 adult mouse primary cardiomyocytes is very mature (Li et al., 2021). However, the survival time of adult mouse cardiomyocytes is very short under CIH or IR, which can only survive for 48 h and cannot experience CIH or IR. In addition, it takes at least 72/96 h for cardiomyocytes to successfully overexpress/knockdown Mirt1, and adult mouse primary cardiomyocytes also cannot survive for so long under hypoxic conditions. This is why we did not do *in vitro*. Next, we will design three different schemes to overexpression/knockdown LncRNA Mirt1 *in vivo*, and select the most effective one of them to verify the effect of gain- and loss-of-function of Mirt1 on CIH-exaggerated post-MI cardiac remodeling.

In summary, we conducted a comprehensive analysis of the transcriptomic mechanisms underlying cardiac remodeling after MI when exposed to CIH. Our current data identified potential lncRNAs and microRNAs in the expression profiles and confirmed that Mirt1 can function as a ceRNA for miR-466i-5p to modulate the HIF-1 and PI3K-Akt signaling pathways by further targeting the level of corresponding mRNAs, such as Enah and Maf. Based on these transcriptional analyses, we speculated that Mirt1 and miR-466i-5p may be the most critical transcriptional regulator during cardiac remodeling after MI. Our data provide fundamental research clues to the transcriptomic mechanisms underlying CIH-exaggerated post-MI cardiac remodeling. Further studies are now needed to confirm the regulatory mechanisms that associate Mirt1 and miR-466i-5p with the HIF-1 and PI3K-Akt signaling pathways.

## Data Availability

The datasets presented in this study can be found in online repositories. The names of the repository/repositories and accession number(s) can be found in the article/supplementary material.
